# Potential of circulating miR-29a and miR-142-5p as biomarkers for diabetic nephropathy: a cross-sectional study

**DOI:** 10.3389/fmed.2026.1804671

**Published:** 2026-06-17

**Authors:** Qian Liu, Xuanxuan Jiao, Tongdao Xu, Chuanmei He, Fang Yang, Fumeng Yang

**Affiliations:** 1 Department of Laboratory Medicine, Affiliated Lianyungang Clinical College of Nantong University, Lianyungang, China; 2 Department of Laboratory Medicine, The Second People’s Hospital of Lianyungang Affiliated with Kangda College of Nanjing Medical University, Lianyungang, China; 3 Department of Laboratory Medicine, Affiliated Lianyungang Clinical College of Bengbu Medical University, Lianyungang, China; 4 School of Medicine, Yangzhou Polytechnic University, Yangzhou, China; 5 Department of Endocrinology, The Second People’s Hospital of Lianyungang, Lianyungang, China; 6 Department of Nephrology, The Second People’s Hospital of Lianyungang, Lianyungang, China

**Keywords:** diabetic nephropathy, microRNA, miR-142-5p, miR-29a, qRT-PCR

## Abstract

**Background:**

Diabetic nephropathy (DN), a major microvascular complication of type 2 diabetes mellitus (T2DM), is a leading cause of end-stage renal disease. Early diagnosis remains challenging because conventional biomarkers have limited sensitivity. Circulating microRNAs, including miR-29a and miR-142-5p, have recently emerged as promising diagnostic candidates in several diseases. This study aimed to compare serum levels of miR-29a and miR-142-5p between T2DM patients with and without DN and to evaluate their potential diagnostic utility.

**Methods:**

This cross-sectional, single-center observational study enrolled 164 patients with T2DM and 164 healthy controls. Participants with T2DM were stratified into three subgroups according to urinary albumin-to-creatinine ratio (UACR). Serum miR-29a and miR-142-5p expression levels were measured using quantitative reverse transcription PCR. Clinical parameters were statistically analyzed, and logistic regression and receiver operating characteristic (ROC) curve analyses were performed to assess diagnostic performance.

**Results:**

Serum miR-29a and miR-142-5p levels were significantly elevated in patients with DN and increased progressively with disease severity. Both microRNAs were identified as independent risk factors for DN. A combined model incorporating miR-29a, miR-142-5p, and cystatin C improved diagnostic accuracy, with an area under the curve (AUC) of 0.939.

**Conclusion:**

Elevated serum miR-29a and miR-142-5p levels are strongly associated with DN progression, supporting their potential as non-invasive biomarkers for the diagnosis and risk stratification of patients with T2DM.

## 1 Introduction

Type 2 diabetes mellitus (T2DM) is a common metabolic disorder with substantial public health implications and is frequently complicated by microvascular disease (1–3). Diabetic nephropathy (DN), one of the most prevalent and severe complications of diabetes, imposes a considerable clinical burden. Epidemiological studies estimate that 20%–40% of patients with diabetes develop DN, which is a major cause of end-stage renal disease (ESRD) (4, 5). A global meta-analysis encompassing 13 countries reported a pooled prevalence of DN of 27% (95% confidence interval: 21%, 33%) among patients with T2DM (6). Although the pathogenesis of DN has not been fully elucidated, its characteristic manifestations include persistent proteinuria, glomerulosclerosis, and progressive decline in glomerular filtration rate (GFR) (7). At present, urinary microalbumin is commonly used as a non-invasive biomarker for the identification, monitoring, and prognostic assessment of DN. However, its diagnostic reliability is limited by susceptibility to several confounding factors, including urinary tract infection, hypertension, and strenuous exercise (8). In addition, conventional biochemical markers such as serum urea and creatinine have insufficient sensitivity for detecting early-stage DN (9). Therefore, reliable novel biomarkers for DN are needed. Such biomarkers may facilitate earlier clinical intervention and help attenuate or prevent T2DM-related renal injury.

MicroRNAs (miRNAs) are small non-coding RNA molecules, typically 19–24 nucleotides in length, that regulate gene expression post-transcriptionally by binding to complementary sequences in target mRNAs. These regulatory RNAs often interact with transcription factors within shared signaling pathways to mediate biological functions (10). Typically, miRNAs bind to the 3’-untranslated regions (3’-UTRs) of mRNAs and repress translation without necessarily inducing transcript degradation. Accumulating evidence over the past two decades indicates that miRNAs regulate more than 60% of human protein-coding genes (11). Previous studies have shown that hyperglycemia-induced upregulation of miR-29a in pancreatic beta cells may promote the transition from impaired glucose tolerance to T2DM. Members of the miR-29 family have also been implicated in the onset and progression of DN (12, 13). More recently, mesenchymal stem cell-derived exosomal long non-coding RNA zinc finger E-box-binding homeobox 1 antisense 1 was shown to alleviate DN progression by regulating inflammation and oxidative stress through the miR-142-5p/PTEN signaling axis; in that study, miR-142-5p expression was markedly increased in a rat model of DN (14). These observations, largely derived from animal studies, suggest that both miR-29a and miR-142-5p participate in the pathogenic mechanisms of DN (12–14). Nevertheless, data on their serum expression patterns in patients with human DN remain limited. Therefore, the present study used quantitative reverse transcription polymerase chain reaction (qRT-PCR) to quantify serum miR-29a and miR-142-5p levels in clinical subjects and evaluate their potential utility as diagnostic biomarkers for DN.

## 2 Materials and methods

### 2.1 Participants

A total of 164 hospitalized patients diagnosed with T2DM were enrolled from the Departments of Endocrinology and Nephrology at the Second People’s Hospital of Lianyungang between January 2023 and December 2024. T2DM was diagnosed according to the 2016 American Diabetes Association (ADA) criteria (15). Based on the expert consensus on DN prevention and management, participants were stratified into three groups according to urinary albumin-to-creatinine ratio (UACR) (15): normal albuminuria (NA; UACR < 30 mg/g; *n* = 57), microalbuminuria (MA; 30 mg/g ≤ UACR < 300 mg/g; *n* = 55), and clinical proteinuria (CP; UACR ≥ 300 mg/g; *n* = 52). According to the diagnostic criteria for DN (16), DN was defined as UACR ≥ 30 mg/g. Accordingly, the MA and CP groups were combined into a single DN group for subsequent independent risk-factor and receiver operating characteristic (ROC) curve analyses. In addition, 164 healthy individuals undergoing routine physical examinations at the same hospital during the same period were recruited as the normal control (NC) group.

Exclusion criteria were as follows:

(1) Primary or secondary hypertension, defined as systolic blood pressure (SBP) > 139 mmHg and/or diastolic blood pressure (DBP) > 89 mmHg;

(2) Acute trauma, active inflammation, or any acute or chronic infectious disease;

(3) Known primary or secondary renal disease (e.g., acute or chronic glomerulonephritis, pyelonephritis, nephrotic syndrome, or interstitial nephritis);

(4) Endocrine or metabolic abnormalities (e.g., hyperthyroidism or Cushing’s syndrome);

(5) Acute or chronic hepatic, pulmonary, or cardiovascular disease;

(6) Malignancy or autoimmune disease (e.g., leukemia or systemic lupus erythematosus);

(7) Recent treatment with angiotensin-converting enzyme inhibitors (ACEIs) or angiotensin II receptor blockers (ARBs), such as captopril or irbesartan; and

(8) Use of nephrotoxic agents within the previous month.

The study protocol was approved by the Ethics Committee of the Second People’s Hospital of Lianyungang (approval No. 2022K020). All participants provided written informed consent, and the study was conducted in accordance with the Declaration of Helsinki.

### 2.2 Instruments and reagents

The following laboratory equipment was used: a benchtop high-speed centrifuge (Baiyang Centrifuge Co., Ltd., China), a high-speed refrigerated centrifuge (Eppendorf, Germany), an ultra-low-temperature freezer (Haier, China), a medical-grade low-temperature freezer (Haier, China), an ultrapure water purification system (Millipore, USA), a microplate reader (Thermo Fisher Scientific, USA), a PCR thermal cycler (Applied Biosystems, USA), a qRT-PCR system (Applied Biosystems, USA), and an AU5800 fully automated biochemical analyzer (Beckman Coulter, USA).

Total RNA was extracted using TRIzol reagent (Life Technologies, USA). Chloroform and isopropanol (Sinopharm Chemical Reagent Co., Ltd., China) were used for phase separation and precipitation, respectively. RNase-free DEPC-treated water (Beyotime Biotechnology, China) was used throughout RNA preparation. Reverse transcription and qRT-PCR amplification were performed using commercially available RT-PCR and qRT-PCR kits (Vazyme Biotech Co., Ltd., China). All primers were synthesized by AccuBioTech Co., Ltd., (China).

Biochemical parameters, including urinary microalbumin (mALB), alanine aminotransferase (ALT), aspartate aminotransferase (AST), gamma-glutamyl transferase (GGT), urea, creatinine (Crea), glucose (GLU), total cholesterol (TC), triglycerides (TG), high-density lipoprotein cholesterol (HDL-C), low-density lipoprotein cholesterol (LDL-C), cystatin C, and β2-microglobulin (β2-MG), were measured using assay kits from Beckman Coulter (USA).

### 2.3 Sample collection

All participants were instructed to fast for 8–14 h before sample collection. The following morning, 2 mL of venous blood was collected into tubes containing EDTA-K2 anticoagulant, and 5 mL was collected into plain serum tubes without anticoagulant. Glycated hemoglobin A1c (HbA1c) was measured from EDTA-K2-anticoagulated blood within 4 h. Plain serum samples were centrifuged within 4 h of collection; 1 mL of separated serum was transferred to sterile, nuclease-free Eppendorf tubes and stored at −80 °C for subsequent batch analysis of serum miR-29a and miR-142-5p expression. The remaining serum was immediately used for biochemical testing, and estimated glomerular filtration rate (eGFR) was calculated using the full age spectrum equation (17). Serum indices, including hemolysis, lipemia, and icterus, were assessed during biochemical testing. Only samples without hemolysis, lipemia, or icterus were included in subsequent analyses.

Approximately 10 mL of fresh first-morning midstream urine was also collected from each participant. Urinary biochemical indicators were measured once, and all measurements were completed within 6 h to preserve sample integrity and ensure result reliability.

### 2.4 Extraction of total serum RNA

#### 2.4.1 Sample preparation

Frozen serum samples were thawed on ice and thoroughly mixed by vortexing. A 200 μL aliquot of serum was transferred into a sterile, nuclease-free Eppendorf (EP) tube. Subsequently, 1 mL of TRIzol reagent was added, and the mixture was homogenized by repeated pipetting.

#### 2.4.2 Lysis and incubation

Samples were incubated on ice in the dark for 10–15 min to ensure complete cell lysis and RNA stabilization.

#### 2.4.3 Phase separation

Chloroform (200 μL) was added to each tube, followed by vigorous inversion for 1–2 min. Samples were incubated on ice for 10 min and centrifuged at 14,000 rpm for 10 min at 4 °C using a refrigerated high-speed centrifuge.

#### 2.4.4 RNA precipitation

The upper aqueous phase was carefully transferred to a new nuclease-free tube. An equal volume of isopropanol was added, mixed by gentle inversion, and incubated on ice for 10 min. Samples were then centrifuged at 14,000 rpm for 10 min at 4 °C to precipitate RNA.

#### 2.4.5 RNA washing

The supernatant was discarded, and the RNA pellet was washed with 1 mL of 75% ethanol. The pellet was resuspended by gentle pipetting and centrifuged again at 14,000 rpm for 10 min at 4 °C.

#### 2.4.6 RNA elution

After discarding the ethanol, the RNA pellet was air-dried at room temperature until no visible liquid remained. The RNA was then dissolved in ≥20 μL of DEPC-treated water, with the volume adjusted according to pellet size.

#### 2.4.7 Evaluation of RNA quality

After dilution of 2 μL of RNA sample, 0.1% DEPC water was used as the blank. A UV spectrophotometer was then used to determine RNA concentration and optical density ratios (OD260/OD280 and OD260/OD230). Acceptable RNA quality was defined as an OD260/OD280 ratio of 1.8–2.1 and an OD260/OD230 ratio > 1.0. Under these conditions, RNA preparations were considered suitable for immediate use in downstream assays or for storage at −80 °C until further analysis.

### 2.5 Quantitative analysis of miRNA

#### 2.5.1 RT-PCR

The stem-loop reverse transcription method was used to synthesize complementary DNA (cDNA) from target miRNAs. Gene-specific stem-loop reverse primers were custom-designed and synthesized by AccuBioTech Co., Ltd., (Hunan, China). A master mix was prepared according to the number of samples, and the reverse-transcription reaction system was configured as follows: RNase-free double-distilled water (ddH_2_O), 4 μL; 2x RT Mix, 10 μL; HiScript II Enzyme Mix, 2 μL; Oligo(dT)23VN (50 μM), 1 μL; random hexamers (50 ng/μL), 1 μL; and total RNA template, 2 μL.

The reaction mixture was gently mixed, dispensed into nuclease-free EP tubes, and subjected to reverse transcription in a thermal cycler under the following conditions: 25 °C for 5 min, 50 °C for 15 min, and 85 °C for 2 min. After completion of the reverse-transcription program, the synthesized cDNA was stored at −20 °C until quantitative PCR analysis.

#### 2.5.2 Real-time quantitative polymerase chain reaction

Real-time quantitative polymerase chain reaction is a fluorescence-based technique that enables real-time monitoring of PCR amplification by incorporating specific fluorescent probes into the reaction system. Fluorescence accumulation is directly proportional to target-sequence amplification, allowing quantitative analysis of gene expression through appropriate data-processing algorithms. In this study, TaqMan MGB probes (Novizan, China) were used to quantify the target miRNAs.

(1) Primer sequences

The following primer sequences (5′→3′) were used for the detection of target miRNAs:

hsa-miR-142-5p-F: CCACGGCATAAAGTAGAAAGCA; hsa-miR-142-5p-stem: GTCGTATCCAGTGCAGGGTCCGAGGTATT CGCACTGGATACGACAGTAGT; hsa-miR-29a-stem: GTCGTA TCCAGTGCAGGGTCCGAGGTATTCGCACTGGATACGACTA ACCG; hsa-miR-29a-F: CGACCTAGCACCATCTGAAATC

(2) Reaction system configuration

Each 20 μL qRT-PCR reaction mixture contained the following components: 2x TaqMan Universal PCR Master Mix, 10 μL; forward primer (Primer 1), 0.4 μL; reverse primer (Primer 2), 0.4 μL; template cDNA, 2 μL; and nuclease-free ddH_2_O, 7.2 μL.

(3) Plate loading and pre-run preparation

Reaction mixtures were loaded into a 96-well qPCR plate at 20 μL per well. All reactions were performed in triplicate. The plate was briefly centrifuged at 2,000 rpm for 1 min at room temperature to eliminate bubbles and ensure even reagent distribution.

(4) qRT-PCR cycling conditions

The amplification was carried out using a real-time PCR instrument under the following cycling conditions:

Initial denaturation was performed at 95 °C for 30 s, followed by 40 amplification cycles of 95 °C for 10 s and 60 °C for 30 s. The dissociation stage consisted of 95 °C for 15 s, 60 °C for 60 s, and 95 °C for 15 s. For each sample, three replicate wells were prepared, along with a blank control in which DEPC water was added in place of the sample. The mean value of the three replicate measurements was used as the final result for calculation.

(5) Verification of amplification efficiency

Serial dilutions of cDNA were prepared to construct standard curves and assess the amplification efficiency of the primer/probe sets. The amplification efficiencies for miR-29a, miR-142-5p, and U6 ranged from 90% to 110%, with R2 values > 0.99.

#### 2.5.3 Relative quantification of serum miR-142-5p and miR-29a

The relative expression levels of serum miR-142-5p and miR-29a were determined using the comparative Cq (2^−ΔCq^) method, with U6 small nuclear RNA as the internal reference. ΔCq values were calculated by subtracting the Cq value of U6 from that of each target miRNA (ΔCq = Cq_miRNA_ − Cq_U6_).

U6 was not subjected to serial dilution or standard-curve construction in this experiment; it was used only as an endogenous control to normalize miR-142-5p and miR-29a expression. Therefore, the obtained values reflect relative rather than absolute quantification of the target miRNAs.

### 2.6 Statistical analyses

All statistical analyses were performed using SPSS version 19.0 (IBM Corp., Armonk, NY, USA). The normality of continuous variables was assessed using the Kolmogorov-Smirnov test. For non-normally distributed data, non-parametric tests were applied: the Mann-Whitney U test for comparisons between two groups and the Kruskal-Wallis H test for comparisons among three or more groups. When significant between-group differences were detected, pairwise comparisons were performed using Dunn’s *post-hoc* test. Categorical variables were analyzed using the chi-square (χ^2^) test. Binary logistic regression was used to identify independent predictors of DN. The diagnostic performance of candidate biomarkers was evaluated using ROC curves, and the area under the curve (AUC) was calculated to quantify predictive accuracy. All tests were two-sided, and *P* < 0.05 was considered statistically significant.

## 3 Results

### 3.1 Demographic and laboratory characteristics of study participants

A total of 164 patients with T2DM and 164 healthy controls were included. The demographic and biochemical characteristics of all participants are summarized in Table 1. No statistically significant differences were observed between the T2DM and control groups in age, sex, body mass index (BMI), smoking status, ALT, AST, or GGT (*P* > 0.05). However, compared with healthy controls, patients with T2DM had significantly higher SBP, DBP, fasting blood glucose (FBG), urea, Crea, TC, TG, HDL-C, LDL-C, β2-MG, cystatin C, UACR, miR-29a, and miR-142-5p levels (*P* < 0.05), whereas eGFR was significantly lower.

**TABLE 1:** Conventional and laboratory indicators of all participants.

Parameters	Control group (*n* = 164)	T2DM group (*n* = 164)	*P*-value
Age (years)	60 (54, 67)	60 (51, 71)	0.0843
Sex (male/female)	105/59	113/51	0.4130
BMI (kg/m^2^)	24.85 (24.00, 26.60)	25.00 (24.30, 25.60)	0.3907
SBP (mm Hg)	125 (122, 127)	130 (126, 134)	<0.0001
DBP (mm Hg)	82 (79, 85)	84 (80, 86)	<0.0001
Smoking status (yes/no)	42/122	60/104	0.0579
Course of disease (years)	–	8.2 (6.5, 9.8)	–
FBG (mmol/L)	5.20 (4.94, 5.58)	8.47 (7.08, 10.80)	<0.0001
HbA1c (%)	4.92 ± 0.52	7.78 ± 0.85	<0.0001
ALT (U/L)	21 (16, 29)	19 (14, 27)	0.0739
AST (U/L)	21 (17, 25)	20 (16, 25)	0.1389
GGT (U/L)	24 (17, 40)	24 (16, 36)	0.3401
Urea (mmol/L)	5.10 (4.40, 5.90)	6.45 (5.03, 8.30)	<0.0001
Crea (μmol/L)	63 (56, 73)	70 (57, 85)	0.0010
TC (mmol/L)	4.93 (4.46, 5.03)	5.62 (5.23, 6.15)	<0.0001
TG (mmol/L)	1.36 (0.94, 1.91)	1.70 (1.08, 2.43)	0.0018
HDL-C (mmol/L)	1.10 (1.01, 1.20)	1.23 (1.11, 1.34)	<0.0001
LDL-C (mmol/L)	2.98 (2.72, 3.05)	3.52 (3.22, 4.00)	<0.0001
β2-MG (mg/L)	1.38 (1.21, 1.64)	2.00 (1.55, 2.63)	<0.0001
eGFR (mL/min/1.73 m^2^)	110 (105, 118)	99 (92, 109)	<0.0001
Cystatin C (mg/L)	0.76 (0.66, 0.84)	0.99 (0.75, 1.53)	<0.0001
UACR (mg/g)	7.60 (4.70, 13.85)	61.92 (17.33, 455.30)	<0.0001
miR-29a*	1.10 (0.94, 1.47)	2.88 (2.16, 3.64)	<0.0001
miR-142-5p*	0.97 (0.69, 1.24)	2.31 (1.53, 2.74)	<0.0001

T2DM, type 2 diabetes mellitus; BMI, body mass index; SBP, systolic blood pressure; DBP, diastolic blood pressure; FBG, fasting blood glucose; HbA1c, glycated hemoglobin A1c; ALT, alanine aminotransferase; AST, aspartate aminotransferase; GGT, gamma-glutamyl transferase; Urea, urea; Crea, creatinine; TC, total cholesterol; TG, triglycerides; HDL-C, high-density lipoprotein cholesterol; LDL-C, low-density lipoprotein cholesterol; β2-MG, β2-microglobulin; eGFR, estimated glomerular filtration rate; UACR, urinary albumin-to-creatinine ratio; miR-29a, microRNA-29a; miR-142-5p, microRNA-142-5p; *Indicates the relative expression level normalized to U6 as the internal reference.

### 3.2 Conventional and laboratory indicators among T2DM patients grouped by UACR

**FIGURE 1:**
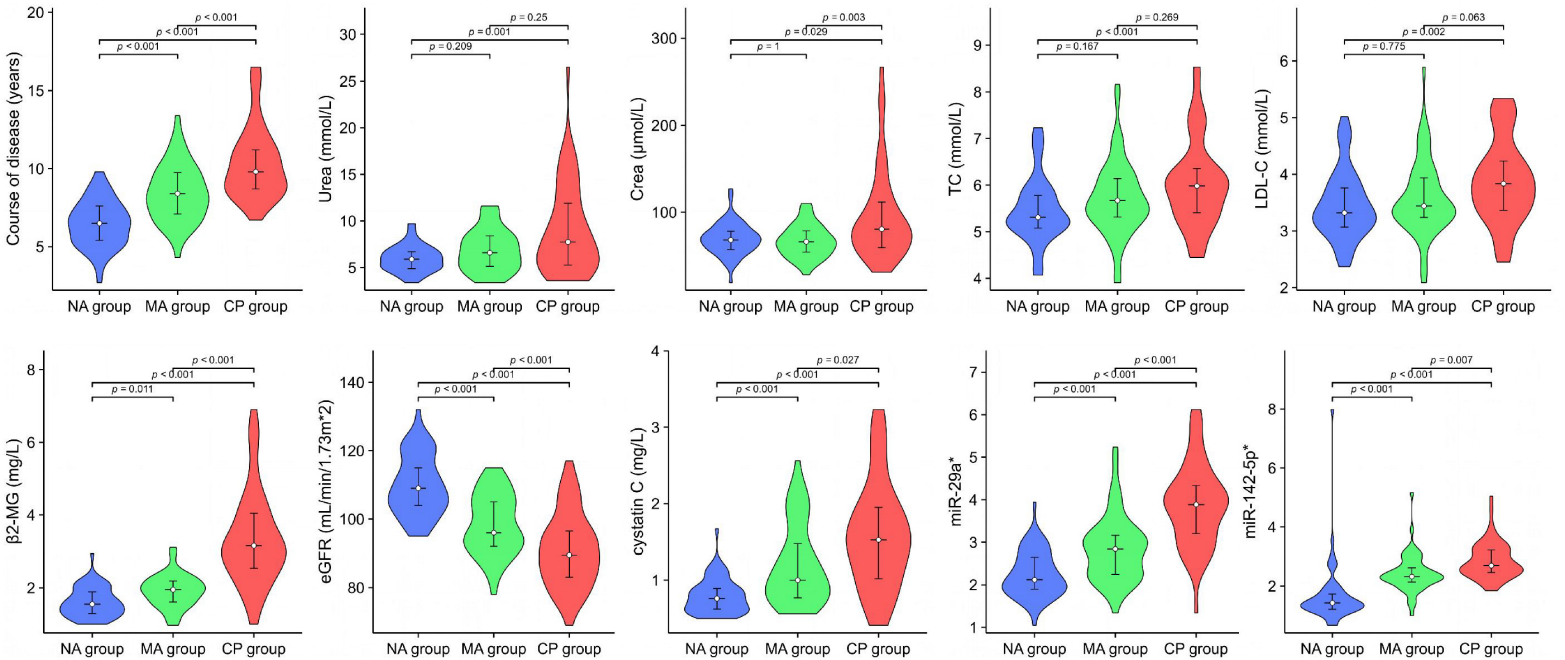
Comparison of biochemical indices among patients with T2DM. Scatter plot showing the levels of each indicator among the NA, MA, and CP groups. Error bars indicate the median and interquartile range. *Indicates the relative expression level normalized to U6 as the internal reference.

Participants with T2DM were categorized into three subgroups according to UACR: NA, MA, and CP. No statistically significant differences were found among the three subgroups in age, sex, BMI, SBP, DBP, FBG, HbA1c, ALT, AST, GGT, TG, or HDL-C (*P* > 0.05). In contrast, significant between-group differences were observed for disease duration, urea, Crea, TC, LDL-C, β2-MG, eGFR, cystatin C, miR-29a, and miR-142-5p. Specifically, as DN severity increased, disease duration and β2-MG, cystatin C, miR-29a, and miR-142-5p levels gradually increased, whereas eGFR declined. Detailed comparative results are presented in Table 2, and the corresponding trends are illustrated in Figure 1. Results of the detection of miR-29a and miR-142-5p are presented in Supplementary Table 1. The raw results are provided in the Supplementary material.

**TABLE 2:** Conventional and laboratory indicators among T2DM patients grouped by UACR.

Indicators	T2DM			*P*-value
	NA group (UACR < 30, *n* = 57)	MA group (30 ≤ UACR < 300, *n* = 55)	CP group (UACR ≥ 300, *n* = 52)	
UACR (mg/g)	10.26 (5.68, 19.11)	71.17 (50.00, 127.30)	772.40 (468.20, 1180.00)	<0.0001
Age (years)	59 (54, 66)	64 (55, 72)	65 (55, 80)	0.0618
Sex (male/female)	38/19	39/16	36/16	0.8874
BMI (kg/m^2^)	24.60 (24.10, 25.30)	25.10 (24.30, 25.70)	25.30 (24.30, 26.00)	0.1587
SBP (mm Hg)	128 (126, 130)	130 (126, 134)	130 (125, 135)	0.2403
DBP (mm Hg)	84 (82, 85)	85 (82, 86)	84 (79, 86)	0.7556
Smoking status (yes/no)	16/41	21/34	23/29	0.2068
Course of disease (years)	6.5 (5.4, 7.6)	8.4 (6.7, 9.8)	9.8 (8.7, 11.2)	<0.0001
FBG (mmol/L)	8.27 (6.98, 9.44)	8.65 (7.13, 11.78)	8.67 (7.02, 12.18)	0.0950
HbA1c (%)	7.19 ± 0.39	7.31 ± 0.55	7.44 ± 0.95	0.0670
ALT (U/L)	21 (14, 29)	21 (15, 31)	18 (12, 21)	0.0623
AST (U/L)	20 (17, 23)	21 (17, 29)	18 (15, 24)	0.2072
GGT (U/L)	22 (17, 35)	27 (16, 36)	25 (16, 39)	0.8284
Urea (mmol/L)	5.90 (4.90, 6.80)	6.60 (5.10, 8.40)	7.75 (5.23, 12.10)	0.0019
Crea (μmol/L)	68 (57, 78)	66 (54, 79)	81 (58, 115)	0.0025
TC (mmol/L)	5.31 (5.08, 5.83)	5.67 (5.31, 6.16)	5.98 (5.36, 6.39)	0.0015
TG (mmol/L)	1.70 (1.01, 2.20)	1.39 (0.98, 2.52)	1.88 (1.27, 2.80)	0.1120
HDL-C (mmol/L)	1.23 (1.12, 1.33)	1.22 (1.10, 1.34)	1.23 (1.10, 1.34)	0.7455
LDL-C (mmol/L)	3.32 (3.07, 3.80)	3.44 (3.24, 3.95)	3.84 (3.35, 4.25)	0.0022
β2-MG (mg/L)	1.55 (1.29, 1.91)	1.95 (1.61, 2.19)	3.16 (2.54, 4.10)	<0.0001
eGFR (mL/min/1.73 m^2^)	109 (104, 114)	96 (92, 105)	90 (83, 98)	<0.0001
Cystatin C (mg/L)	0.76 (0.61, 0.90)	1.00 (0.77, 1.54)	1.53 (1.01, 1.97)	<0.0001
miR-29a*	2.12 (1.89, 2.65)	2.84 (2.21, 3.16)	3.89 (3.20, 4.35)	<0.0001
miR-142-5p*	1.43 (1.21, 1.77)	2.32 (2.11, 2.65)	2.70 (2.46, 3.27)	<0.0001

T2DM, type 2 diabetes mellitus; NA group, normal albuminuria group; MA group, microalbuminuria group; CP group, clinical proteinuria group; UACR, urinary albumin-to-creatinine ratio; BMI, body mass index; SBP, systolic blood pressure; DBP, diastolic blood pressure; FBG, fasting blood glucose; HbA1c, glycated hemoglobin A1c; ALT, alanine aminotransferase; AST, aspartate aminotransferase; GGT, gamma-glutamyl transferase; Urea, urea; Crea, creatinine; TC, total cholesterol; TG, triglycerides; HDL-C, high-density lipoprotein cholesterol; LDL-C, low-density lipoprotein cholesterol; β2-MG, β2-microglobulin; eGFR, estimated glomerular filtration rate; miR-29a, microRNA-29a; miR-142-5p, microRNA-142-5p; *Indicates the relative expression level normalized to U6 as the internal reference.

### 3.3 Identification of independent risk factors for DN

To ensure the reliability of the binary logistic regression model, multicollinearity diagnostics were performed using SPSS version 19.0. TC and LDL-C each had a variance inflation factor (VIF) of 6.7 and a tolerance of 0.15, whereas all other variables had VIF values < 2 and tolerance values > 0.2. Because LDL-C was considered the main contributor to TC elevation, LDL-C was selected for inclusion in the binary logistic regression model. The following variables were entered as independent variables: age, sex, BMI, SBP, DBP, smoking status, disease duration, urea, Crea, LDL-C, β2-MG, eGFR, cystatin C, miR-29a, and miR-142-5p. DN status (DN vs. non-DN) served as the dependent variable. Because cystatin C levels were skewed, natural logarithmic transformation was applied before analysis. Regression analysis identified serum cystatin C, miR-29a, and miR-142-5p as independent predictors of DN. The corresponding odds ratios (ORs) and 95% confidence intervals (CIs) were as follows: log-transformed cystatin C, OR = 9.969 (95% CI: 1.272–78.139, *P* = 0.029); miR-29a, OR = 3.985 (95% CI: 1.363–11.656, *P* = 0.012); and miR-142-5p, OR = 15.719 (95% CI: 4.077–60.599, *P* < 0.001). These findings indicate that elevated serum cystatin C, miR-29a, and miR-142-5p levels are significant independent risk factors for DN (Table 3).

**TABLE 3:** Binary logistic regression analysis of risk factors for DN.

Independent variable	B	SE	Wals χ^2^	*P*-value	OR (95% CI)
Age	0.057	0.033	2.956	0.086	1.059 (0.992–1.131)
Sex	0.387	0.767	0.255	0.614	1.473 (0.328–6.618)
BMI	0.315	0.280	1.267	0.260	1.371 (0.792–2.373)
SBP	0.132	0.103	1.629	0.202	1.141 (0.932–1.396)
DBP	−0.048	0.158	0.092	0.762	0.953 (0.699–1.300)
Smoking status	−0.339	1.024	0.109	0.741	0.713 (0.096–5.300)
Course of disease	0.405	0.221	3.349	0.067	1.499 (0.972–2.313)
Urea	0.106	0.204	0.272	0.602	1.112 (0.746–1.659)
Crea	−0.016	0.019	0.717	0.397	0.984 (0.947–1.022)
LDL-C	−0.713	0.916	0.605	0.437	0.490 (0.081–2.954)
β2-MG	0.809	0.671	1.451	0.228	2.245 (0.602–8.370)
eGFR	−0.044	0.035	1.530	0.216	0.957 (0.893–1.026)
Cystatin C	2.300	1.051	4.791	0.029	9.969 (1.272–78.139)
miR-29a	1.383	0.548	6.377	0.012	3.985 (1.363–11.656)
miR-142-5p	2.755	0.688	16.010	0.000	15.719 (4.077–60.599)
Constant	−29.301	17.089	2.940	0.086	0.000

BMI, body mass index; SBP, systolic blood pressure; DBP, diastolic blood pressure; Urea, urea; Crea, creatinine; LDL-C, low-density lipoprotein cholesterol; β2-MG, β2-microglobulin; eGFR, estimated glomerular filtration rate; miR-29a, microRNA-29a; miR-142-5p, microRNA-142-5p; B, regression coefficients; SE, standard error; OR, odds ratio; CI, confidence interval.

### 3.4 Diagnostic performance of cystatin C, miR-29a, and miR-142-5p in DN

**FIGURE 2:**
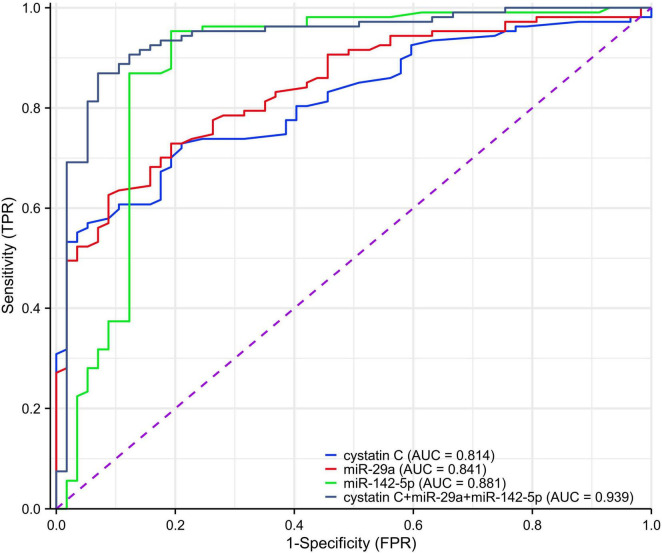
Receiver operating characteristic (ROC) curves for serum cystatin C, miR-29a, and miR-142-5p in patients with DN. When cystatin C alone was used, the area under the ROC curve was 0.814 (95% CI: 0.751–0.878; *P* < 0.0001; cutoff value: 0.905 mg/L). When miR-29a alone was used, the area under the ROC curve was 0.841 (95% CI: 0.781–0.900; *P* < 0.0001; cutoff value: 2.945). When miR-142-5p alone was used, the area under the ROC curve was 0.881 (95% CI: 0.813–0.950; *P* < 0.0001; cutoff value: 1.845). When the three indicators were combined, the area under the ROC curve was 0.939 (95% CI: 0.897–0.981; *P* < 0.0001).

According to the established diagnostic criteria, DN was defined as UACR ≥ 30 mg/g (16). Because cystatin C, miR-29a, and miR-142-5p were identified as independent risk factors, ROC curve analysis was performed to evaluate their diagnostic value. All three biomarkers showed significant diagnostic efficacy in distinguishing T2DM patients with DN from those without DN. When assessed individually, diagnostic performance ranked as follows: miR-142-5p (AUC = 0.881), miR-29a (AUC = 0.841), and cystatin C (AUC = 0.814). Notably, the combination of the three biomarkers substantially improved diagnostic accuracy, achieving an AUC of 0.939. Internal validation of the AUC for the combined model (cystatin C + miR-29a + miR-142-5p) was performed using bootstrap resampling with 1,000 iterations. Cross-validation yielded a mean AUC of 0.917 (95% CI: 0.881–0.953), confirming model robustness and a low risk of overfitting. Detailed diagnostic parameters for each biomarker are summarized in Table 4 and illustrated in Figure 2.

**TABLE 4:** Diagnostic value of cystatin C, miR-29a, and miR-142-5p in DN.

Indicators	Cut-off values	AUC (95% CI)	Sen (%)	Spec (%)	Accu (%)	PPV (%)	NPV (%)	Youden index (%)
Cystatin C	0.905	0.814 (0.751–0.878)	72.90	78.95	75.00	86.67	60.81	51.85
miR-29a	2.945	0.841 (0.781–0.900)	62.62	91.23	72.56	93.06	56.52	53.85
miR-142-5p	1.845	0.881 (0.813–0.950)	95.33	80.70	90.24	90.27	90.20	76.03
Cystatin C + miR-29a + miR-142-5p	–	0.939 (0.897–0.981)	86.92	92.98	89.02	95.88	79.10	79.90

AUC, area under the ROC curve; CI, confidence interval; Sen, sensitivity; Spec, specificity; Accu, accuracy; PPV, positive predictive value; NPV, negative predictive value.

## 4 Discussion

Accumulating evidence suggests that circulating miRNAs are dysregulated in various pathological conditions, highlighting their potential as biomarkers for disease diagnosis and monitoring, including in DN (18–20). Recent bioinformatics analyses have identified miR-29a as a potential diagnostic and therapeutic target in DN-related renal cell carcinoma (21). In addition, systematic reviews have consistently reported elevated miR-29a expression in individuals with DN, suggesting its involvement in the molecular pathogenesis of the disease (22). Similarly, miR-142-5p has been proposed as a biomarker for gestational diabetes, and its downstream target PTEN, a key regulator of autophagy, has been shown to play an important role in DN progression (23, 24). However, studies investigating serum miR-29a and miR-142-5p expression levels in patients with DN remain limited. To address this gap, the present study used qRT-PCR to measure serum miR-29a and miR-142-5p levels in healthy controls, patients with uncomplicated T2DM, and T2DM patients with DN. The aim was to characterize their expression profiles and assess their potential associations with the onset and progression of DN.

In the present study, patients with T2DM had significantly higher blood pressure and more pronounced dyslipidemia than normoglycemic controls. These metabolic alterations reflect cardiovascular and lipid-regulatory disturbances commonly associated with diabetes and may contribute to disease progression and related complications. Serum miR-29a, miR-142-5p, β2-MG, and cystatin C levels were also significantly higher in patients with T2DM than in healthy individuals. This finding is consistent with the meta-analysis by Zhu et al. (23), which reported significant upregulation of miR-29a in patients with T2DM and suggested its potential as a circulating biomarker. Zhang et al. (25) further demonstrated that miR-142-5p regulates endothelial cell function by targeting Nrf2 and modulating the Nrf2/NLRP3 signaling pathway. Their study showed that suppression of miR-142-5p reduced interleukin-1β (IL-1β) levels in a mouse model of type 1 diabetes, suggesting a potential anti-inflammatory effect of miR-142-5p inhibition in diabetic vascular complications. Similarly, Wang et al. (26) reported significantly higher serum β2-MG levels in patients with T2DM than in healthy controls. In a cross-sectional study of 100 patients with T2DM, Mahashabde et al. (27) found elevated cystatin C levels in 91% of participants. Collectively, these findings underscore the potential roles of miR-29a, miR-142-5p, β2-MG, and cystatin C in the pathophysiology of T2DM and support their utility as candidate biomarkers for disease monitoring and risk stratification.

Patients with T2DM were classified into three subgroups according to UACR levels: NA, MA, and CP. Based on the diagnostic criteria for DN (28), the MA and CP groups were defined as DN cases. The T2DM subgroups differed significantly in disease duration and in urea, Crea, TC, LDL-C, β2-MG, cystatin C, eGFR, miR-29a, and miR-142-5p levels. Notably, disease duration and β2-MG, cystatin C, miR-29a, and miR-142-5p levels increased progressively with worsening DN severity, whereas eGFR showed the opposite trend. These observations are consistent with the study by Chien et al. (29), which reported substantially higher miR-29a levels in patients with overt proteinuria than in those with microalbuminuria and demonstrated elevated miR-29a expression in DN progressors compared with non-progressors. Similarly, Sun et al. (30) emphasized the important role of macrophage-driven inflammation in miR-29a-mediated diabetes progression. Wang et al. (26) also demonstrated that serum β2-MG levels increase significantly during the transition from T2DM to early-stage DN. Consistent with our findings, Yu et al. (31) observed statistically significant increases in serum cystatin C across subgroups as DN advanced. In the present study, serum creatinine and LDL-C were significantly elevated only in patients with clinical proteinuria, suggesting limited sensitivity for early DN detection. We observed markedly elevated serum miR-29a and miR-142-5p levels in patients with DN, and these levels were positively associated with disease severity. To explore the potential molecular mechanisms underlying these findings, we integrated evidence from the existing literature. Regarding fibrosis regulation, Yang et al. (32) demonstrated that miR-29a-3p alleviates DN-related renal fibrosis by suppressing DNMT3A/3B expression and modulating the Wnt/β-catenin and JAK/STAT signaling pathways. This finding suggests that the upregulation of miR-29a observed in our study may represent a compensatory anti-fibrotic response, although its long-term effects require further validation. With respect to the PTEN signaling pathway, Chen et al. (33) showed that miR-142-5p is significantly upregulated in DN models and exacerbates renal fibrosis by directly targeting PTEN, activating the PI3K/AKT/mTOR pathway, and consequently reducing autophagy. In addition, a systematic review by Ma et al. (34) summarized key signaling networks involved in diabetic renal fibrosis, including the TGF-β/Smad, JAK/STAT, Wnt/β-catenin, and PI3K/Akt pathways, and emphasized their central roles in extracellular matrix deposition and epithelial-mesenchymal transition. Taken together, this mechanistic evidence suggests that the elevated serum miR-29a and miR-142-5p levels observed in our study may be associated with DN onset and progression through fibrosis-related pathways and the PTEN/PI3K/AKT signaling axis.

Binary logistic regression analysis further showed that cystatin C, miR-29a, and miR-142-5p were independent risk factors for DN. Supporting this finding, Liang et al. (35) reported that both miR-29a and miR-142-5p independently predicted T2DM, impaired fasting glucose, and insulin resistance. ROC curve analysis was then performed to evaluate diagnostic performance. All three biomarkers–cystatin C, miR-29a, and miR-142-5p–showed significant diagnostic value for DN. Among them, miR-142-5p had the highest diagnostic accuracy, followed by miR-29a and cystatin C. Notably, the combined model achieved superior diagnostic efficacy compared with any single biomarker, suggesting improved clinical utility for DN risk prediction. In line with this result, Kang et al. (36) reported in a cross-sectional study that cystatin C was significantly associated with renal function decline (OR = 2.255, *P* = 0.008) and showed excellent diagnostic performance for predicting renal impairment (AUC = 0.974). Together, these findings reinforce that miR-29a and miR-142-5p are upregulated in patients with DN and highlight their potential as predictive biomarkers for clinical evaluation and early DN detection.

Several limitations should be acknowledged. First, all participants were recruited from a single center; therefore, regional and population-specific biases may limit the generalizability of the findings. Second, owing to the cross-sectional design and the absence of longitudinal follow-up, no prospective power calculation was performed at the study design stage, and the prognostic value of miR-29a and miR-142-5p for DN progression could not be evaluated. Third, although this study systematically examined the associations between serum miR-29a and miR-142-5p levels and DN, the molecular mechanisms by which these miRNAs contribute to disease pathogenesis remain unclear. Fourth, U6 may not be an optimal normalizer for serum miRNA levels because its stability in circulation remains controversial. Future studies should therefore incorporate more stable exogenous spike-in controls, such as cel-miR-39, MIR2911, or other validated controls, to confirm these findings. In light of these limitations, future research should include multicenter, large-scale case-control studies combined with animal models of DN to clarify the mechanisms of action of miR-29a and miR-142-5p. Such efforts may help define their biological functions and advance their clinical translation as diagnostic markers and therapeutic targets for DN.

## 5 Conclusion

In conclusion, serum miR-29a and miR-142-5p levels are strongly associated with the development and progression of DN, suggesting that these miRNAs may serve as novel non-invasive biomarkers for DN diagnosis. Nevertheless, prospective multicenter studies with larger sample sizes are needed to validate these findings, clarify the underlying molecular mechanisms, and establish their clinical utility in the early detection and management of DN.

## Data Availability

The original contributions presented in this study are included in this article/Supplementary material, further inquiries can be directed to the corresponding author.

## Supplementary material

Supplementary Table 1 Raw detection results of miR-29a and miR-142-5p.

## References

[1] Liu Y Ma K Zhang A Cui Y Zhao H Li X et al. Increased fatty acid-binding protein 4 levels are associated with the risk of developing retinopathy in type 2 diabetes mellitus patients. *Diabetes Metab*. (2025) 51:101653. 10.1016/j.diabet.2025.101653 40254126

[2] Dariya SS Maheshwari A Viswanathan V Virmani AK Aslam M Modi A et al. Assessment of the awareness of risk factors and current behavior among individuals with type 2 diabetes mellitus in India: a cross-sectional study. *Cureus*. (2025) 17:e80512. 10.7759/cureus.80512 40225536 PMC11993084

[3] Ran L Han Y Zhaohu H Hailin S. Correlation between triglyceride-glucose index and microvascular complications in patients with early- onset of type 2 diabetes mellitus. *Endocrinol Diabetes Metab*. (2025) 8:e70027. 10.1002/edm2.70027 39946246 PMC11824366

[4] Thomas MC Brownlee M Susztak K Sharma K Jandeleit-Dahm KA Zoungas S et al. Diabetic kidney disease. *Nat Rev Dis Primers*. (2015) 1:15018. 10.1038/nrdp.2015.18 27188921 PMC7724636

[5] Xue L Zhang Y Zhang Q. The relationship between advanced glycation end products, metabolic metrics, HbA1c, and diabetic nephropathy. *Front Endocrinol (Lausanne)*. (2025) 16:1468737. 10.3389/fendo.2025.1468737 40123890 PMC11925793

[6] Fenta ET Eshetu HB Kebede N Bogale EK Zewdie A Kassie TD et al. Prevalence and predictors of chronic kidney disease among type 2 diabetic patients worldwide, systematic review and meta-analysis. *Diabetol Metab Syndr*. (2023) 15:245. 10.1186/s13098-023-01202-x 38012781 PMC10683270

[7] Lin DN Li D Peng MM Yang H Lin ZZ Ye EL et al. Elevated waist-to-hip ratio, as an abdominal obesity index, predicts the risk of diabetic kidney injury. *World J Diabetes*. (2025) 16:101384. 10.4239/wjd.v16.i4.101384 40236864 PMC11947918

[8] Lv N Jia L Liu F Cheng L Liu F Kuang J et al. Elevated circulating homocysteine concentrations delayed nerve conduction velocity and increase the risk of diabetic kidney disease in patients with type 2 diabetes. *Front Endocrinol (Lausanne)*. (2024) 15:1451758. 10.3389/fendo.2024.1451758 39722814 PMC11668604

[9] Sharon Rose P Shashidhar KN Munilakshmi U Meghanath M. Fluoride enigma in end stage renal disease. *Bioinformation*. (2024) 20:998–1001. 10.6026/973206300200998 39917236 PMC11795493

[10] Woo SJ Kim Y Jung H Lee JJ Hong JY. MicroRNA 148a suppresses tuberculous fibrosis by targeting NOX4 and POLDIP2. *Int J Mol Sci*. (2022) 23:2999. 10.3390/ijms23062999 35328424 PMC8954251

[11] Beylerli O Beeraka NM Gareev I Pavlov V Yang G Liang Y et al. MiRNAs as noninvasive biomarkers and therapeutic agents of pituitary adenomas. *Int J Mol Sci*. (2020) 21:7287. 10.3390/ijms21197287 33023145 PMC7583927

[12] Bagge A Clausen TR Larsen S Ladefoged M Rosenstierne MW Larsen L et al. MicroRNA-29a is up-regulated in beta-cells by glucose and decreases glucose-stimulated insulin secretion. *Biochem Biophys Res Commun.* (2012) 426:266–72. 10.1016/j.bbrc.2012.08.082 22940552

[13] Mansouri E Orazizadeh M Mard SA Gorji AV Rashno M Fakhredini F. Therapeutic effect of kidney tubular cells-derived conditioned medium on the expression of microRNA-377, microRNA-29a, aquapurin-1, biochemical, and histopathological parameters following diabetic nephropathy injury in rats. *Adv Biomed Res*. (2022) 11:119. 10.4103/abr.abr_375_21 36798914 PMC9926036

[14] Liu DF Hu FL. [Exosomal lncRNA ZEB1-AS1 secreted from mesenchymal stem cells attenuates diabetic nephropathy by regulating miR-142-5p/PTEN axis]. *Chin J Endocrinol Metab*. (2023) 39:695–703. 10.3760/cma.j.cn311282-20221210-00686

[15] American Diabetes Association. Standards of medical care in diabetes-2019: classification and diagnosis of diabetes. *Diabetes Care*. (2019) 42:S13–28. 10.2337/dc19-S002 30559228

[16] Chen H Du P Jiang T Li Y Li Y Liu Y et al. Identification of potential biomarkers for diabetic nephropathy via UPLC-MS/MS-based metabolomics. *Front Endocrinol (Lausanne)*. (2025) 16:1581691. 10.3389/fendo.2025.1581691 40958907 PMC12433848

[17] Liu Q Hang H Xu T Zhang Y Yang F Wang X et al. Establishment of reference intervals for estimated glomerular filtration rate in apparently healthy adults based on the full age spectrum equation: a single-centre study. *Ann Clin Biochem*. (2025) 62:174–83. 10.1177/00045632241306060 39631771

[18] Yin R Zhang Y Fang X Zhang Y Miao R Yao Y et al. Discovering diabetes complications-related microRNAs: meta-analyses and pathway modeling approach. *BMC Med Genomics*. (2025) 8:86. 10.1186/s12920-025-02144-1 40369506 PMC12079836

[19] Balkan II Shahzadi A Sönmez H Oktan B Umar MI Mete B et al. Longitudinal analysis of hsa-miR-3163, hsa-miR-124-3p, hsa-miR-548c-3p, and hsa-miR-27a-3p as prognostic biomarkers in HIV-infected patients. *Front Immunol*. (2025) 16:1565068. 10.3389/fimmu.2025.1565068 40396180 PMC12089146

[20] Zhao W Wen JX Niu Y Yan L Wang MY Jiao W et al. Exosomal miR-182-5p is a potential diagnostic marker for malignant pleural effusion. *Transl Lung Cancer Res*. (2025) 14:1138–48. 10.21037/tlcr-2024-1205 40386717 PMC12082201

[21] Dong Y Zhai W Xu Y. Bioinformatic gene analysis for potential biomarkers and therapeutic targets of diabetic nephropathy associated renal cell carcinoma. *Transl Androl Urol*. (2020) 9:2555–71. 10.21037/tau-19-911 33457229 PMC7807343

[22] Assmann TS Recamonde-Mendoza M de Souza BM Bauer AC Crispim D. MicroRNAs and diabetic kidney disease: systematic review and bioinformatic analysis. *Mol Cell Endocrinol*. (2018) 477:90–102. 10.1016/j.mce.2018.06.005 29902497

[23] Zhu H Leung SW. MicroRNA biomarkers of type 2 diabetes: evidence synthesis from meta-analyses and pathway modelling. *Diabetologia*. (2023) 66:288–99. 10.1007/s00125-022-05809-z 36269347 PMC9807484

[24] Bai X Zhou Y Chen P Yang M Xu J. MicroRNA-142-5p induces cancer stem cell-like properties of cutaneous squamous cell carcinoma via inhibiting PTEN. *J Cell Biochem*. (2018) 119:2179–88. 10.1002/jcb.26379 28857248

[25] Zhang R Niu S Rong Z Li F Ni L Di X et al. A potential target for diabetic vascular damage: high glucose-induced monocyte extracellular vesicles impair endothelial cells by delivering miR-142-5p. *Front Bioeng Biotechnol*. (2022) 10:913791. 10.3389/fbioe.2022.913791 35615474 PMC9124888

[26] Wang D Wang W Xiang S Xia C Zhang Y Zhang L. The application value of multimodal ultrasound imaging technology in the prediction of early-stage type 2 diabetic kidney disease. *Sci Rep*. (2025) 15:12520. 10.1038/s41598-025-97151-8 40216906 PMC11992222

[27] Mahashabde ML Chauhan RS Paidi SKR Sriram PJ. Study of cystatin C and renal resistive index in type 2 diabetes mellitus patients to detect early diabetic kidney disease. *Ann Afr Med*. (2026) 25:424–30. 10.4103/aam.aam_124_25 40758098 PMC13056279

[28] Molitch ME DeFronzo RA Franz MJ Keane WF Mogensen CE Parving HH et al. Nephropathy in diabetes. *Diabetes Care*. (2004) 27:S79–83. 10.2337/diacare.27.2007.s79 14693934

[29] Chien HY Chen CY Chiu YH Lin YC Li WC. Differential microRNA profiles predict diabetic nephropathy progression in Taiwan. *Int J Med Sci*. (2016) 13:457–65. 10.7150/ijms.15548 27279796 PMC4893561

[30] Sun Y Zhou Y Shi Y Zhang Y Liu K Liang R et al. Expression of miRNA-29 in pancreatic β cells promotes inflammation and diabetes via TRAF3. *Cell Rep*. (2021) 34:108576. 10.1016/j.celrep.2020.108576 33406428

[31] Yu C Qu Z Zhou S Zhao R Bao L. Serum Homocysteine (Hcy), Cystatin C (Cys-C), and Urine Microalbumin (mAlb) are critical for early diagnosis of diabetic nephropathy. *Am J Transl Res*. (2025) 17:7408–25. 10.62347/DMQO5042 41112996 PMC12531566

[32] Yang Y Chen Y Tang JY Chen J Li GQ Feng B et al. MiR-29a-3p inhibits fibrosis of diabetic kidney disease in diabetic mice via downregulation of DNA methyl transferase 3A and 3B. *World J Diabetes*. (2025) 16:93630. 10.4239/wjd.v16.i4.93630 40236856 PMC11947916

[33] Chen J Cui Y Zhang N Yao X Wang Z Yang L. Oleanolic acid attenuated diabetic mesangial cell injury by activation of autophagy via miRNA-142-5p/PTEN signaling. *Cytotechnology*. (2019) 71:925–33. 10.1007/s10616-019-00335-0 31410746 PMC6787128

[34] Ma Y Wang J Fan J Jia H Li J. Interrelation of natural polyphenol and fibrosis in diabetic nephropathy. *Molecules*. (2024) 30:20. 10.3390/molecules30010020 39795078 PMC11722366

[35] Liang YZ Dong J Zhang J Wang S He Y Yan YX. Identification of neuroendocrine stress response-related circulating microRNAs as biomarkers for type 2 diabetes mellitus and insulin resistance. *Front Endocrinol (Lausanne).* (2018) 9:132. 10.3389/fendo.2018.00132 29643835 PMC5882838

[36] Kang Y Jin Q Zhou M Zheng H Li X Li A et al. Diagnostic value of serum TGF-β1 and CysC in type 2 diabetic kidney disease: a cross-sectional study. *Front Med (Lausanne)*. (2025) 12:1529648. 10.3389/fmed.2025.1529648 40291021 PMC12021808

